# Low-protein vegetarian diet does not have a short-term effect on blood acid–base status but raises oxygen consumption during submaximal cycling

**DOI:** 10.1186/1550-2783-9-50

**Published:** 2012-11-26

**Authors:** Enni-Maria Hietavala, Risto Puurtinen, Heikki Kainulainen, Antti A Mero

**Affiliations:** 1 Department of Biology of Physical Activity, University of Jyväskylä, P.O. Box 35 (VIV), Jyväskylä, FIN-40014, Finland

**Keywords:** Nutrition, Acid–base balance, Aerobic performance

## Abstract

**Background:**

Acid–base balance refers to the equilibrium between acids and bases in the human body. Nutrition may affect acid–base balance and further physical performance. With the help of PRAL (potential renal acid load), a low-protein vegetarian diet (LPVD) was designed to enhance the production of bases in body. The aim of this study was to investigate if LPVD has an effect on blood acid–base status and performance during submaximal and maximal aerobic cycling.

**Methods:**

Nine healthy, recreationally active men (age 23.5 ± 3.4 yr) participated in the study and were randomly divided into two groups in a cross-over study design. Group 1 followed LPVD for 4 days and group 2 ate normally (ND) before performing a cycle ergometer test. The test included three 10-min stages at 40, 60 and 80% of VO_2_max. The fourth stage was performed at 100% of VO_2_max until exhaustion. After 10–16 days, the groups started a second 4-day diet, and at the end performed the similar ergometer test. Venous blood samples were collected at the beginning and at the end of both diet periods and after every stage cycled.

**Results:**

Diet caused no significant difference in venous blood pH, strong ion difference (SID), total concentration of weak acids (A_tot_), partial pressure of CO_2_ (pCO_2_) or HCO_3_^-^ at rest or during cycling between LPVD and ND. In the LPVD group, at rest SID significantly increased over the diet period (38.6 ± 1.8 vs. 39.8 ± 0.9, p=0.009). Diet had no significant effect on exercise time to exhaustion, but VO_2_ was significantly higher at 40, 60 and 80% of VO_2_max after LPVD compared to ND (2.03 ± 0.25 vs. 1.82 ± 0.21 l/min, p=0.035; 2.86 ± 0.36 vs. 2.52 ± 0.33 l/min, p<0.001 and 4.03 ± 0.50 vs. 3.54 ± 0.58 l/min, p<0.001; respectively).

**Conclusion:**

There was no difference in venous blood acid–base status between a 4-day LPVD and ND. VO_2_ was increased during submaximal cycling after LPVD suggesting that the exercise economy was poorer. This had no further effect on maximal aerobic performance. More studies are needed to define how nutrition affects acid–base balance and performance.

## Background

For normal functioning of the human body, there must be equilibrium between acids and alkali in body fluids [1]. Almost all function of enzymes and cells is dependent on the acid–base balance [2]. The acidity or alkalinity of body fluids is usually expressed by pH, which is affected by hydrogen ion concentration ([H^+^). In arteries, normal pH is 7.4. During acidosis there is an excess of hydrogen ions and pH is below 7.4, whereas during alkalosis hydrogen ions are lost and pH is above 7.4. Regulation mechanisms of the acid–base balance try to maintain pH in body fluids strictly between 7.37 and 7.43 [2]. According to the physicochemical approach of Peter Stewart, there are three independent variables that determine the hydrogen ion concentration and, thus, pH of body fluids: strong ion difference (SID), total concentration of weak acids (A_tot_) and partial pressure of carbon dioxide (pCO_2_) [3]. The approach of Stewart is a more versatile way to explore the acid–base balance than the traditional, CO_2_-centered Henderson-Hasselbalch equation [4].

SID is the difference between strong cations and anions and can be calculated as: SID (mEq/l) = ([Na^+^ + [K^+^) - ([Cl^-^ + [Lac^-^) [5]. When SID increases, [H^+^ decreases according to the rule of electroneutrality. SID is usually slightly positive, but fluids of the body cannot be electrically charged. The necessary negative charge comes from pCO_2_ and A_tot_. When the production of CO_2_ exceeds the removal of CO_2_ in the metabolism of cells, pCO_2_ increases and causes a rise in [H^+^. A_tot_ is mainly proteins (mainly albumin) and phosphates and through them the rule of electroneutrality is fulfilled. If there is a change in one or more independent variable, [H^+^ changes as a consequence [3].

It is known that nutrition has an effect on acid–base balance, that is, acid load of the human body can be changed via nutrition [6]. It can be evaluated via PRAL (potential renal acid load) whether a certain foodstuff increases the production of acids or alkali in the body [6,7]. PRAL can be calculated for 100 g of foodstuff as: PRAL (mEq/100 g) = 0.49 × protein (g/100 g) + 0.037 × phosphorous (mg/100 g) - 0.021 x potassium (mg/100 g) - 0.026 × magnesium (mg/100 g) - 0.013 × calcium (mg/100 g) [7]. A foodstuff with negative PRAL is more alkali than acid forming. For example, fruits and vegetables contain lots of potassium that is a base-forming cation along with magnesium and calcium. Conversely, meat, cheese and cereal products have a positive PRAL and they enhance the production of acids. All protein-rich foodstuffs contain amino acids methionine and cysteine that are acid forming, so nutrition rich in protein and poor in alkali-forming foodstuff increases the acid load of the body [6].

The acid–base balance has an effect on physical performance [8]. Even physical activity of moderate intensity causes metabolic changes, which affect the acid–base balance both in skeletal muscles and other tissues [3]. Maintenance of high alkalinity in extracellular fluids enables faster H^+^ removal from the muscle cell and muscle fatigue caused by increased acidosis is delayed [8]. Enhanced acid buffering capacity seems to improve both high-intensity anaerobic [9,10] and aerobic [11] capacity. NaHCO_3_ is a useful ergogenic aid to increase the [HCO_3_^-^ and buffering capacity of the blood [12], but performance can be improved by dietary means as well [13,14]. It has been observed that protein-rich nutrition combined with a low intake of carbohydrate may cause acidosis and have a negative influence on performance [13]. In one study, for example, low-protein (9.4 ± 1.8%) and high-carbohydrate (65.5 ± 9.8%) diet obeyed for 4 days resulted in higher plasma pH and [HCO_3_^-^ prior to the exercise test compared to high-protein (25.3 ± 4.1%) and low-carbohydrate (10.1 ± 6.8%) diet and resulted in a longer time to exhaustion during cycling at 100% of VO_2_max (345 ± 187 s vs. 221 ± 58 s) [14]. In another study, the use of a plant-based nutrient supplement for 14 days increased the pH of urine, which indicates that the acid load of the body was decreased [15]. These findings provide rationale to study the effects of a low-protein vegetarian diet on acid–base balance and physical performance.

According to our knowledge, there are no previous studies where the PRAL method is used to evaluate the quality of food for the investigation of the effect of nutrition on aerobic performance in humans. Thus, the purpose of this study was to explore if a low-protein vegetarian diet, which was designed with the help of PRAL to enhance the production of bases, has an effect on acid–base balance in men. Moreover, the study was planned to determine whether the possible changes in venous blood acid–base status influence performance or fuel selection during submaximal and maximal cycling. It was hypothesized that a diet low in protein and rich in alkali-producing vegetables and fruits may have the potential to alter the blood acid–base status and, thus, enable higher aerobic capacity and influence fuel selection during exercise.

## Methods

### Subjects

Nine healthy, recreationally active men volunteered for the study and signed an informed consent. Subjects were students of University of Jyväskylä and were exercising recreationally (e.g. walking, jogging, cycling, resistance training). Subjects who were obese (body mass index above 30), were training for competitive purposes, were using any medication or had any food allergy were excluded from the study. Ethical approval for the study was obtained from the University’s Ethics Committee and the study followed the declaration of Helsinki.

### Pre-testing

Before the actual experimental cross-over design, VO_2_max and maximal workload of the subjects were measured (measurement 1, M1). Before M1 the subjects followed their normal diet and kept food diaries for 4 days, thus, the eating and drinking habits of the subjects were checked to be in accordance with general dietary guidelines. On the fifth day, the subjects performed M1, which was an incremental VO_2_max test performed on a mechanically braked cycle ergometer (Ergomedic 839E, Monark Exercise AB, Vansbro, Sweden). The workload was initially 75 W and was increased by 25 W every 2 min until exhaustion. The pedaling frequency was sustained at 60 rpm throughout the test. Before the ergometer test, height, weight and body mass index (BMI) of the subjects were determined. For the estimation of body fat percentage, a 4-point skinfold method was used. Thicknesses of biceps, triceps, subscapular and suprailiac skinfolds were measured and standard equations of Durnin & Womersley [16] were used for the determination of fat percentage.

### Experimental design

**Figure 1:**
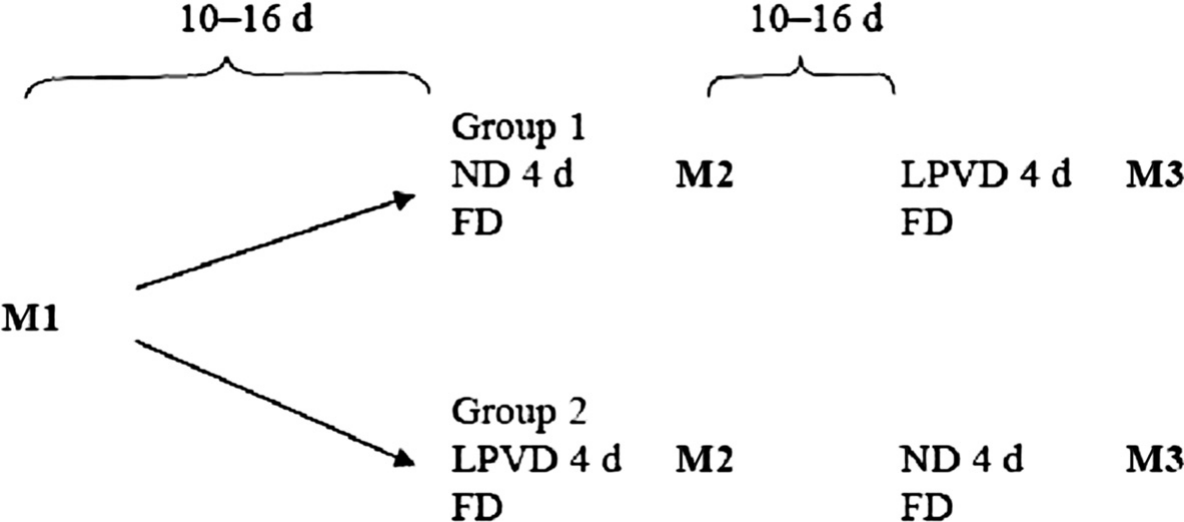
**The study design.** FD= food diary, ND= normal diet, LPVD= low-protein vegetarian diet, M1= VO_2_max cycle ergometer test, M2 and M3= Cycle ergometer tests after the LPVD and ND.

The study design is presented in Figure 1. After M1, subjects were randomly divided into two groups. Group 1 (n=5) followed a normal diet (ND) first and then a low-protein vegetarian diet (LPVD). Group 2 (n=4) followed LPVD first and then ND. 10–16 days after M1, subjects came to the laboratory at 8 or 10 am after a 12-hour overnight fast and fasting blood samples (PREdiet) from a fingertip capillary and an antecubital vein were drawn. The last meal before PREdiet was consistent with the normal diet of the subjects. Starting from the PREdiet sample, the subjects followed either LPVD or ND and kept food diaries for 4 days. On the 5th day they completed the second measurement (M2). On the morning of M2, after a 12-hour overnight fast, fasting blood samples (POSTdiet) were drawn at the same time as PREdiet. The last meal before POSTdiet was consistent with the diet followed during the 4 days (either LPVD or ND). A light breakfast, which was consistent with the assigned diet, was eaten thereafter. After a rest of 30 min, resting blood samples were drawn once more (PREtest). The subjects started M2 by a 5-min warm-up followed by a 4-min break before the actual test started. According to the results of M1, workloads for M2 and M3 (measurement 3) were determined. In M2 and M3, the subjects cycled 3 × 10 min at 40, 60 and 80% of VO_2_max and finally at 100% of VO_2_max until exhaustion. For every subject the workload was increased by 50 or 75 W in every stage. There were 4-min breaks after each 10-min cycling stage during which blood samples were collected (Stage 1−4).

After M2 was completed, the subjects were allowed to eat according to their normal dietary habits without keeping a food diary. 10–16 days after M2, the subjects started the second 4-day diet and on the 5th day completed M3. M3 was similar to M2, but before M3 the groups changed the diets. All the blood samples were drawn at the same time in the morning as during the first diet period.

The subjects were allowed to exercise moderately during the diet periods. However, during the last 24 hours before every fasting blood sample the subjects were advised to minimize their physical activity and strenuous exercise was not allowed. The subjects reported their physical activity during both diet periods along with food diaries. Thus, it was controlled that the instructions concerning physical activity were obeyed.

### PRAL and the diets

LPVD was designed with the help of PRAL to enhance the production of alkali in the body. A PRAL value of every foodstuff used in LPVD was calculated according to an equation that takes into account the contents of certain nutrients per 100 g of foodstuff, their intestinal absorption rates, grade of dissociation of phosphate at pH 7.4 and the ionic valence of magnesium and calcium. The equation is as follows: PRAL (mEq/100 g) = 0.49 × protein (g/100 g) + 0.037 × phosphorous (mg/100 g) - 0.021 x potassium (mg/100 g) - 0.026 x magnesium (mg/100 g) - 0.013 × calcium (mg/100 g) [7]. The PRAL values were calculated according to the nutrient contents that were taken from the Finnish Food Composition Database (Fineli, Finnish National Institute of Health and Welfare). When the PRAL value is below 0 the foodstuff is assumed to enhance the production of alkali in the body, and when it is above 0 the foodstuff increases the production of acids. Foodstuffs used during LPVD were chosen according to their PRAL value so that the diet would enhance the alkali production as much as possible. However, the general dietary guidelines were taken into account as well.

The subjects were given exact instructions how to realize LPVD. All the days during the vegetarian diet were similar and the diet mainly contained vegetables and fruits. The use of grain and dairy products was very limited. The subjects were not allowed to eat e.g. meat, cheese, eggs or bread at all during the 4 days. During both LPVD and ND the subjects were instructed to eat according to their energy needs and they reported the amount of foods eaten in a food diary.

### Blood sampling and analysis

For the analysis of acid–base balance, Li-heparinized whole blood samples (200 μl) from a fingertip capillary were analyzed immediately after sampling for pH, lactate, HCO_3_^-^ and pCO_2_. For the determination of pH the direct ISE (ion selective electrolyte) in vitro test was used. Lactate was analyzed quantitatively by the enzymatic and amperometric in vitro test. PCO_2_ was analyzed by the membrane amperometric method. HCO_3_^-^ was determined computationally (Nova Biomedical STAT Profile pHOX Plus L Blood Gas Analyzator, Nova Biomedical, Waltham, MA, USA). Whole blood samples (4 ml) from the antecubital vein were collected to Venosafe gel tubes and analyzed for sodium, potassium and chloride by the direct ISE in vitro test (Ion Selective Microlyte Analyzer, Kone Instruments, Espoo Finland). Whole protein content of plasma and serum albumin were analyzed spectrophotometrically by the Biuret method (Shimadzu CL 720 Micro-Flow Spectrophotometry, Shimadzu Co., Kyoto, Japan).

Glucose was determined from the Li-heparinized fingertip samples (200 μl) quantitatively by the enzymatic and amperometric in vitro test (Nova Biomedical STAT Profile pHOX Plus L Blood Gas Analyzer). Non-esterified free fatty acids (FFA) and triglycerides (TG) were analyzed from the antecubital whole blood sample (4 ml). The blood samples were drawn in vacuum tubes and were centrifuged for 10 min at 3500 rpm. The serum was separated and FFA and TG were then analyzed by the spectrophotometric and enzymatic method. For the determination of FFA, NEFA C-kit was used (Shimadzu CL 720 Micro-Flow Spectrophotometry).

During cycling, the gaseous exchange was measured using Sensor Medics Breath Gas Analyzator (Vmax series 229, California, USA). The device was calibrated before every measurement. VO_2_, VCO_2_, RQ and VE were determined as a mean from the final 30 seconds of every stage. Heart rate was measured by a Polar heart rate monitor (Polar Electro Oy, Kempele, Finland).

SID and A_tot_ were calculated as follows: SID (mEq/l) = ([Na^+^ + [K^+^) - ([Cl^-^ + [Lac^-^) [3], A_tot_ (mEq/l) = 2.43 × [P_tot_ (g/dl) [17].

Food diaries were analyzed for energy, protein, carbohydrate, fat, phosphorous, potassium, magnesium and calcium intake by the Nutri-Flow software (Flow-Team Oy, Oulu, Finland, 2012). The daily PRAL during LPVD and ND were calculated as the overall PRAL per one day according to the actual intake of relevant nutrients.

### Statistical analysis

All the variables were analyzed by SPSS 14.0 for Windows software. The resting blood samples (PREdiet and POSTdiet), the gaseous values, and the nutrient intake values were compared by paired t-test. Variables from the blood samples of M2 and M3 (Stage1–4) were compared to the resting blood sample of the same day (POSTdiet) between the two groups (ND vs. LPVD) with repeated measures ANOVA (2 group × 5 time). If there was a difference between the groups the analysis was continued with paired t-test.

## Results

### Subjects

All nine subjects completed the study design. Subjects were 23.5 ± 3.4 years old (mean ± SD). Their weight measured during pre-testing was 76.7 ± 7.4 kg and height 1.79 ± 0.06 m. BMI of the subjects was 24.0 ± 1.8 and the body fat percentage was 15.6 ± 3.0%. In the incremental VO_2_max test (M1) the exhaustion occurred at 25 ± 2.7 min and VO_2_max of the subjects was 4.10 ± 0.44 l/min.

### Diets

There was a significant difference between the daily PRAL during LPVD and ND (−117 ± 20 vs. 3.2 ± 19, p<0.000). During LPVD subjects consumed 1151 ± 202 g fruits and vegetables whereas during ND the intake of fruits and vegetables was 354 ± 72 g (p<0.000). Energy and nutrient contents of LPVD and ND are presented in Table 1. Energy intake was significantly lower during LPVD compared to ND (2400 ± 338 kcal vs. 2793 ± 554 kcal, p=0.033). During LPVD, the intake of protein was 10.1 ± 0.26% and during ND 17.6 ± 3.0% of the total energy intake (p=0.000). The intake of carbohydrates was significantly higher during LPVD compared to ND (58.7 ± 2.4% vs. 49.8 ± 5.4%, p=0.003). As well, the amount of fat differed between LPVD and ND (24.7 ± 2.3% vs. 28.1 ± 3.1%, p=0.015). In spite of lower energy intake during LPVD there was no difference in the weight of the subjects compared to ND (75.6 ± 7.9 kg vs. 76.2 ± 7.6 kg).

**Table 1:** Energy and nutrient content of normal diet (ND) and low-protein vegetarian diet (LPVD)

		**ND**	**LPVD**
**PRAL (mEq/d)**		3.2 ± 19	−117 ± 20***
**Energy (kcal/d)**		2792 ± 554	2400 ± 338*
**Protein**	**(g/d)**	122 ± 29	61 ± 8.9***
	**(g/kg/d)**	1.59 ± 0.28	0.80 ± 0.11***
	**(%)**	17.6 ± 3.0	10.1 ± 0.26***
**CHO**	**(g/d)**	348 ± 80	349 ± 51
	**(g/kg/d)**	4.58 ± 0.93	4.63 ± 0.61
	**(%)**	49.8 ± 5.4	58.7 ± 2.4**
**Fat**	**(g/d)**	87 ± 20	66 ± 11**
	**(g/kg/d)**	1.14 ± 0.20	0.88 ± 0.13**
	**(%)**	28.1 ± 3.1	24.7 ± 2.3*

*= p<0.05; **= p<0.01; ***= p<0.001.

### Acid–base balance

Diet had no significant effect on venous blood pH (Table 2). There were no significant differences between the diets in SID, A_tot_, pCO_2_ or HCO_3_^-^at rest or during exercise (Tables 2 and 3). The only significant change caused by nutrition was that SID was significantly higher after LPVD compared to before the diet (PREdiet vs. POSTdiet: 38.6 ± 1.8 mEq/l vs. 39.8 ± 0.9 mEq/l, p=0.009).

**Table 2:** **Plasma pH and [HCO**_**3**_^**-**^**] at rest and during cycle ergometer tests**

**Sample**	**pH**		**HCO**_**3**_^**-**^**(mmol/l)**	
	**ND**	**LPVD**	**ND**	**LPVD**
**PREdiet**	7.467 ± 0.039	7.448 ± 0.028	33.6 ± 8.7	32.2 ± 6.0
**POSTdiet**	7.455 ± 0.028	7.454 ± 0.025	32.0 ± 5.5	31.9 ± 3.9
**PREtest**	7.466 ± 0.030	7.459 ± 0.015	32.9 ± 6.3	32.6 ± 4.5
**Stage1**	7.470 ± 0.029	7.473 ± 0.036	31.0 ± 3.1	31.7 ± 4.2
**Stage2**	7.459 ± 0.028	7.457 ± 0.031	28.6 ± 2.3	20.8 ± 3.3
**Stage3**	7.378 ± 0.039*	7.368 ± 0.029**	20.8 ± 3.3**	19.9 ± 2.2***
**Stage4**	7.326 ± 0.076*	7.336 ± 0.03***	16.7 ± 2.5**	18.4 ± 2.4***

ND= normal diet.

LPVD= low-protein vegetarian diet.

PREdiet= a fasting blood sample taken in the morning before the start of ND or LPVD (day 1).

POSTdiet= a fasting blood sample taken in the morning after a 4-day ND or LPVD (day 5).

PREtest= a resting blood sample taken 30 min after a breakfast, before the cycle ergometer test (day 5).

Stage1–4= blood samples taken after 10-min cycling at 40, 60 and 80% of VO_2_max and after the maximal stage (at 100% of VO_2_max until exhaustion).

POSTdiet vs. Stage1–4 *= p<0.05; **= p<0.01; ***= p<0.001.

**Table 3:** Independent variables of acid–base balance at rest and during cycle ergometer tests

**Sample**	**SID (mEq/l)**		**A**_**tot**_**(mEq/l)**		**pCO**_**2**_**(mmHg)**	
	**ND**	**LPVD**	**ND**	**LPVD**	**ND**	**LPVD**
**PREdiet**	38.6 ± 1.8	38.6 ± 1.8	18.5 ± 0.8	18.3 ± 0.6	6.07 ± 1.29	6.13 ± 1.09
**POSTdiet**	39.4 ± 1.2	39.8 ± 0.9^#^	18.1 ± 1.0	18.1 ± 1.0	6.05 ± 0.82	5.98 ± 0.64
**PREtest**	38.8 ± 1.5	38.5 ± 1.2*	18.1 ± 0.8	18.1 ± 1.0	5.98 ± 0.95	6.05 ± 0.89
**Stage1**	38.0 ± 1.1	37.9 ± 0.6**	18.8 ± 0.9	18.9 ± 0.5	5.60 ± 0.38	5.72 ± 0.97
**Stage2**	35.7 ± 1.0*	35.3 ± 1.7**	19.3 ± 0.8**	19.1 ± 0.8**	5.30 ± 0.28	5.27 ± 0.57
**Stage3**	30.6 ± 1.6**	29.5 ± 2.2***	20.2 ± 1.0***	20.1 ± 1.0**	4.61 ± 0.38*	4.55 ± 0.41**
**Stage4**	29.6 ± 3.5**	29.1 ± 2.8***	20.4 ± 1.5**	20.2 ± 1.0***	4.23 ± 0.66*	4.51 ± 0.56**

ND= normal diet.

LPVD= low-protein vegetarian diet.

PREdiet= a fasting blood sample taken in the morning before the start of ND or LPVD (day 1).

POSTdiet= a fasting blood sample taken in the morning after a 4-day ND or LPVD (day 5).

PREtest= a resting blood sample taken 30 min after a breakfast, before the cycle ergometre test (day 5).

Stage1–4= blood samples taken after 10-min cycling at 40, 60 and 80% of VO_2_max and after the maximal stage (at 100% of VO_2_max until exhaustion).

PREdiet compared to POSTdiet ^#^= p<0.05.

POSTdiet vs. Stage1–4 *= p<0.05; **= p<0.01; ***= p<0.001.

Within each diet group, cycling did cause some statistically significant alterations in the variables of acid–base balance, which are presented in Table 2 and 3. These acute responses were similar between both diets.

### Workload and VO_2_

Workload, heart rate and duration of each stage of M2 and M3 are presented in Table 4. Some subjects were not able to finish the 10-min stage of 80% of VO_2_max. In the LPVD group the duration of the stage was 8.84 ± 1.46 min whereas in ND group it was 8.56 ± 1.87 min. The maximal stage (100% of VO_2_max) which was cycled until exhaustion lasted 1.81 ± 0.80 min in the LPVD group and 2.89 ± 1.91 min in the ND group. However, differences in the durations of these stages were not significant. There were no differences in heart rates between the diet groups.

**Table 4:** Workload, duration and heart rate of every stage during cycle ergometer tests

**Workload** **(% of VO**_**2**_**max)**	**Workload (W)**	**Duration (min)**		**Heart rate (bpm)**	
		**ND**	**LPVD**	**ND**	**LPVD**
40	140 ± 10	10	10	128 ± 15	131 ± 12
60	210 ± 20	10	10	156 ± 16	161 ± 10
80	275 ± 30	8.56 ± 1.87	8.84 ± 1.46	180 ± 15	184 ± 10
100	338 ± 35	2.89 ± 1.91	1.81 ± 0.80	183 ± 11	182 ± 12

ND= normal diet.

LPVD= low-protein vegetarian diet.

**Figure 2:**
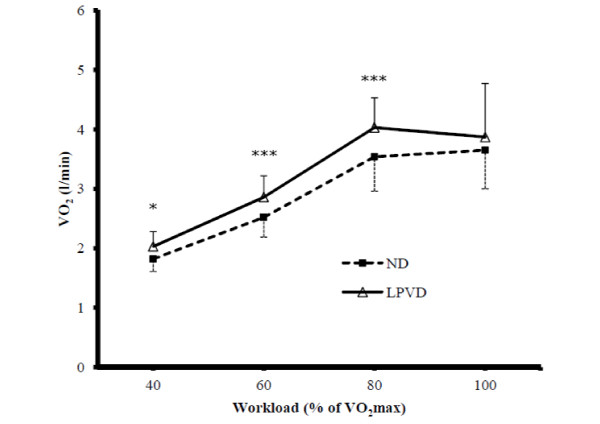
**Oxygen consumption during cycle ergometer tests after normal diet (ND) and low-protein vegetarian diet (LPVD).** *= p<0.05; ***= p<0.001.

The values of VO_2_, VCO_2_, VE and RQ are presented in Table 5. After LPVD, VO_2_ was significantly higher at 40, 60 and 80% of VO_2_max (2.03 ± 0.25 vs. 1.82 ± 0.21 l/min, p=0.035; 2.86 ± 0.36 vs. 2.52 ± 0.33 l/min, p<0.001 and 4.03 ± 0.50 vs. 3.54 ± 0.58 l/min, p<0.001; respectively), but not at 100% of VO_2_max, compared to ND (Figure 2). Also, VCO_2_ differed significantly at all submaximal stages, being higher after LPVD (p=0.011. p=0.009, p=0.010, respectively). VE tended to be higher at all stages after LPVD, but the difference was significant (p=0.009) only at Stage 2. RQ was not different between the diet groups at any point of the cycling.

**Table 5:** **VO**_**2**_**, VCO**_**2**_**, VE and RQ during cycle ergometer tests**

**Work load (% of VO**_**2**_**max)**	**VO**_**2**_**(l/min)**		**VCO**_**2**_**(l/min)**		**VE (l/min)**		**RQ**	
	**ND**	**LPVD**	**ND**	**LPVD**	**ND**	**LPVD**	**ND**	**LPVD**
40	1.82 ± 0.21	2.03 ± 0.25*	1.60 ± 0.2	1.80 ± 0.2**	43.7 ± 5.2	47.7 ± 4.3	0.88 ± 0.03	0.89 ± 0.02
60	2.52 ± 0.33	2.86 ± 0.36***	2.29 ± 0.3	2.59 ± 0.3***	62.9 ± 10	70.7 ± 7.1**	0.91 ± 0.02	0.91 ± 0.03
80	3.54 ± 0.58	4.03 ± 0.50***	3.48 ± 0.7	3.91 ± 0.3**	113 ± 30	130 ± 13	0.98 ± 0.05	0.98 ± 0.04
100	3.65 ± 0.65	3.87 ± 0.90	3.56 ± 0.8	3.62 ± 1.0	131 ± 27	130 ± 40	0.97 ± 0.1	0.95 ± 0.1

ND= normal diet.

LPVD= low-protein vegetarian diet.

*= p<0.05; **= p<0.01; ***= p<0.001.

VO_2_max measured in the first cycle test (M1) was 4.10 ± 0.44 l/min. After LPVD, the highest VO_2_ achieved during Stage 4 was 3.87 ± 0.90, whereas after ND it was 3.65 ± 0.65 l/min. However, none of the VO_2_max values differed significantly from each other.

### Blood carbohydrate and fat metabolites and serum albumin

There were no differences in venous blood lactate, glucose, FFA or TG between the two diet groups at rest or during cycling. At rest, TG decreased significantly (p=0.021) during LPVD (PREdiet vs. POSTdiet). During cycling there were, within each diet group, some statistically significant changes that are presented in Table 6.

**Table 6:** Carbohydrate and fat metabolites and albumin in blood at rest and during cycle ergometer tests

**Sample**	**Lactate (mmol/l)**		**Glucose (mmol/l)**		**FFA (mmol/l)**		**TG (mmol/l)**		**Albumin (g/l)**	
	**ND**	**LPVD**	**ND**	**LPVD**	**ND**	**LPVD**	**ND**	**LPVD**	**ND**	**LPVD**
**PREdiet**	1.6 ± 0.6	1.5 ± 0.5	4.80 ± 0.39	4.83 ± 0.27	0.34 ± 0.21	0.31 ± 0.06	1.18 ± 0.77	1.07 ± 0.30	40.3 ± 2.2	39.4 ± 3.1
**POSTdiet**	1.4 ± 0.5	1.4 ± 0.6	4.95 ± 0.42	4.81 ± 0.21	0.28 ± 0.17	0.35 ± 0.15	0.90 ± 0.23	0.85 ± 0.19^#^	39.1 ± 3.3	41.7 ± 2.0^#^
**PREtest**	2.6 ± 0.7	2.9 ± 1.0	5.16 ± 1.00	6.18 ± 1.28	0.15 ± 0.07	0.22 ± 0.09	0.91 ± 0.23	0.79 ± 0.23	40.3 ± 1.8	39.8 ± 2.9
**Stage1**	2.6 ± 0.9*	2.7 ± 0.9**	4.12 ± 0.44	3.88 ± 0.69	0.13 ± 0.04	0.13 ± 0.05	1.02 ± 0.25	0.82 ± 0.23	40.7 ± 2.4**	41.7 ± 2.8
**Stage2**	4.8 ± 1.2*	5.2 ± 1.9**	4.64 ± 0.63	4.38 ± 0.66	0.18 ± 0.08	0.19 ± 0.07	1.05 ± 0.22	0.89 ± 0.26	43.0 ± 2.5**	42.6 ± 1.2
**Stage3**	10.2 ± 1.6***	11.3 ± 2.1***	5.54 ± 0.79	5.66 ± 0.97	0.22 ± 0.10	0.22 ± 0.06	1.12 ± 0.26*	0.92 ± 0.28	44.8 ± 2.2**	44.7 ± 2.0*
**Stage4**	11.2 ± 3.4**	12.2 ± 2.1***	5.81 ± 0.99	5.21 ± 0.80	0.20 ± 0.10	0.20 ± 0.05	1.16 ± 0.29*	0.93 ± 0.28	44.3 ± 2.7**	44.3 ± 2.7*

ND= normal diet.

LPVD= low-protein vegetarian diet.

PREdiet= a fasting blood sample taken in the morning before the start of ND or LPVD (day 1).

POSTdiet= a fasting blood sample taken in the morning after a 4-day ND or LPVD (day 5).

PREtest= a resting blood sample taken 30 min after a breakfast, before the cycle ergometre test (day 5).

Stage1–4= blood samples taken after 10-min cycling at 40, 60 and 80% of VO_2_max and after the maximal stage (at 100% of VO_2_max until exhaustion).

PREdiet compared to POSTdiet ^#^= p<0.05.

POSTdiet vs. Stage 1–4 *= p<0.05; **= p<0.01; ***= p<0.001.

There were no differences in serum albumin between the diet groups at rest or during cycling. Within LPVD group, albumin increased from 39.4 ± 3.1 g/l (PREdiet) to 41.7 ± 2.0 g/l (POSTdiet) (p=0.032). Within each diet group, cycling caused some statistically significant changes, which are presented in Table 6.

## Discussion

### Main results

The main result of this study was that there was no difference in venous blood acid–base status and its independent or dependent variables between a 4-day LPVD and ND. However, one statistically significant change in acid–base status did occur in the LPVD group, as SID increased by 3.1% over the 4-day diet period. During cycling, the diet composition caused some differences in aerobic energy production, which could be seen in significantly higher VO_2_ and VCO_2_ at every submaximal workload after LPVD compared to ND. This finding had no further effect on maximal aerobic performance.

### Acid–base balance and diets

LPVD did not affect the venous blood acid–base status at rest or during submaximal or maximal cycling compared to ND. The higher protein content of food increases acid production in the body [6], therefore, we hypothesized that lower protein content combined with plentiful consumption of alkalinizing fruits and vegetables would shift the acid–base balance to a more alkaline direction. The PRAL value of every foodstuff consumed in LPVD was under 0, so the diet was clearly designed to enhance the production of alkali in the body. However, during ND subjects ate according to their normal eating habits and PRAL varied from −18.8 to 32.9 mEq/d. Thus, the acid load of ND varied remarkably on an individual level. Changes in blood acid–base status caused by nutrition are generally small, and the large inter-subject variation in PRAL during ND may have masked the possible effects of LPVD on acid–base balance. Moreover, the large variability during ND combined with the small subject group may have made the possible influence of nutrition difficult to detect.

In the present study ND, 17.6 ± 3.0% of the total energy intake (1.59 ± 0.28 g/kg) contained protein and LPVD contained 10.1 ± 0.26% (0.80 ± 0.11 g/kg) protein. The difference was statistically significant, but was not enough to cause changes in acid–base balance. In other studies, the difference has been greater; e.g. there are studies where the protein intakes during high- and low-protein diets have been 25.3 ± 4.1% vs. 9.4 ± 1.8%; 29 ± 4% vs. 10 ± 2% and 33 ± 6% vs. 10 ± 1% [14, 18, 19 respectively]. According to the present and other studies, and in the light of the fact that the protein intake increases the renal capacity to excrete acids [7], it seems that the difference in protein content of the diet must be remarkable to cause differences in acid–base status. Furthermore, the body will normally compensate rapidly for acute changes in acid–base balance to sustain [H^+^ at the optimal level [5]. In the above mentioned studies [14,18,19], for example, pCO_2_ compensated the changes in venous blood pH. As is generally known, pH in body fluids is quite stable, although there are large amount of acids produced constantly in metabolism [1]. It may be that changing diet for only 4 days is not enough to shift acid–base balance to any direction so remarkably that it could be seen in venous blood samples. Since blood pH is strictly regulated, it would be reasonable to also measure urine pH to see if acid load of the body has changed [15].

In the present study we wanted to explore if changing diet from neutral to clearly alkali-producing (instead of two extremes) affects acid–base balance and performance. SID increased by 3.1% during LPVD, which is an encouraging result, but this change was not large enough to cause a detectable change in dependent variables like H^+^ or HCO_3_^-^. Moreover, SID remained at a normal level and did not rise above 40 mmol/l, which can be considered as the lower limit of alkalosis [20]. Nonetheless, our results show that the 4-day diets we compared in this study did not cause a measurable difference in venous blood acid–base status.

### Oxygen consumption and fuel selection during cycling

Nutrition had a statistically significant impact on O_2_ consumption and CO_2_ production during aerobic cycling. After LPVD, both O_2_ and CO_2_ were approximately 13% higher at every submaximal stage of the cycle ergometer test compared to ND. There were no differences in heart rates between the two cycling tests, so the loading for the cardiovascular system and the workload were similar during both tests. When exercising at a constant workload, higher oxygen consumption is usually connected to an increased level of FFA in plasma or increased oxidation of lipids [21]. However, there were no differences in RQ or plasma FFA or TG between the dietary groups. Neither lactate nor glucose contents of plasma were different between the groups, so it is not possible to discuss the changes in the use of substrates in energy production, which could explain the differences in oxygen consumption. On the other hand, in the present study, serum albumin increased during LPVD by 5.8%. This could partially explain the higher oxygen consumption because serum albumin enables a higher rate of FFA transportation to muscle cells [22]. Metabolic acidosis inhibits albumin synthesis [23], so serum albumin content and SID, which both increased during LPVD, refer together to decreased acidosis. More controlled diet interventions should be used in the future to clarify this finding.

In an earlier study by Galloway and Maughan [21], oxygen consumption increased because of alkalosis, when the subjects exercised at 70% of VO_2_max, but there was no difference in RQ. It was discussed that alkalosis would have caused a slight change in the use of substrates, which increased the oxygen consumption, but the change was so small that it could not be seen in RQ. In another study [24], metabolic alkalosis induced by NaHCO_3_ accelerated the increase of VO_2_ at the onset of high-intensity exercise (87% of VO_2_max). However, at a lower intensity (40% of VO_2_max), the alkalosis had no effect on the kinetics of breathing and oxygen consumption. Acidosis may, in turn, reduce the capacity of hemoglobin to bind oxygen and may reduce the oxygen content of the blood [25]. After LPVD, the subjects may have had an increased capacity to transport oxygen in the blood, but because of the lack of measurable change in acid–base status besides the minor change in SID, this is speculation.

It may also be that LPVD increased the need for oxygen, and as a consequence, oxidation of all substrates increased during submaximal cycling, which could explain the lack of changes in RQ. These results suggest that the energy expenditure was greater and cycling economy poorer after LPVD. In the present study insulin-like growth factor 1 (IGF-1) was not measured but according to our recently collected and unpublished data, serum IGF-1 increased during a 7 d high-protein diet and decreased during a 7 d low-protein vegetarian diet. The difference in IGF-1 could be one reason for the difference in oxygen consumption, since lower serum IGF-1 levels may result in poorer exercise economy [26].

In future studies it would be reasonable to control the energy intake of the diets to minimize the effect of difference in caloric intake on performance. However, the subjects were instructed to eat according to their perceived energy needs and they were free to make their own nutritional choices within the given instructions. Although the energy intake was approximately 390 kcal less during LPVD compared to ND, in our opinion, this was not a factor that would cause the difference in VO_2_ between the two diet groups. Furthermore, there was no significant difference in the absolute carbohydrate intake between the diets, so e.g. muscle glycogen content should not have been lower after LPVD. Nonetheless, it seems that the vegetarian diet altered the need for oxygen during submaximal cycling. Since there were no differences in VO_2_max or time until exhaustion between the diet groups the implications of the higher oxygen consumption at submaximal stages for maximal aerobic performance remains unclear.

## Conclusions

A low-protein vegetarian diet followed for 4 days had no acute effect on venous blood acid–base status in young recreationally active men when compared to the normal diet of the subjects. The vegetarian diet increased VO_2_ during submaximal aerobic cycling suggesting that the submaximal cycling economy was poorer after LPVD compared to ND. However, this had no further effect on maximal aerobic performance. According to these results, a low-protein vegetarian diet cannot be recommended as a means to improve submaximal or maximal aerobic performance via acid–base balance as opposed to what was hypothesized. More studies are needed to define how nutrition, its comprehensive composition, and the duration of the diet period affect acid–base balance and performance. More specific measurements should also be used to determine the underlying mechanisms for higher VO_2_ after the low-protein vegetarian diet.

## Competing interests

This study project was funded by University of Jyväskylä, Department of Biology of Physical Activity. The authors declare that they have no competing interests.

## Authors’ contributions

EH (corresponding author) was responsible for the study design, the execution of the measurements, the statistical analysis and the preparation of the manuscript. RP participated in the study design and carried out all the blood sampling and analysis. HK helped in interpretation of data and revised the manuscript. AM supervised the study design, the implementation of the measurements and the drafting and revising the manuscript. All authors read and approved the final manuscript.
